# To what extent did African countries prepare mechanisms to ensure transparency and accountability of the COVID-19 extra-budgetary funds?

**DOI:** 10.7189/jogh.12.03071

**Published:** 2022-11-08

**Authors:** Brendan Kwesiga, Hiba Boujnah, James Avoka Asamani, Juliet Nabyonga-Orem

**Affiliations:** 1 World Health Organization, Kenya country office; Universal health coverage life course cluster; Nairobi Kenya; 2 Africa Centres for Disease Control and Prevention (Africa CDC) – Ethiopia Addis Ababa; 3 World Health Organization, Regional office for Africa, Universal health coverage life course cluster Harare – Zimbabwe; 4 Centre for Health Professions Education, Faculty of Health Sciences, North-West University

The COVID-19 pandemic presented an unprecedented crisis and, in such cases, the need to err on the side of over-preparedness is justified. In all ways – scale and speed in response efforts (no regrets policy), as opposed to a wait-and-see approach, is the preferred option. The World Bank estimated the required additional funding for COVID-19 preparedness and response in sub-Saharan Africa over a period of 250 days to be in the range of US$23.54 billion – $53 billion depending on the social distancing measures employed [1]. Countries employed several mechanisms to finance the COVID-19 response, including reallocating funds away from non-priority areas, releasing supplementary budgets, and creating of special funds [2]. We reviewed the mechanisms used to ensure transparency and accountability of the COVID-19 extra-budgetary funds in African countries. We focused on three parameters: 1) the presence and nature of the management and oversight structures, 2) spending, and 3) accountability modalities.

## MEASURES TAKEN TO FINANCE THE COVID-19 PANDEMIC

As shown in **Table 1**, extra-budgetary funds whose sources of funding, management & oversight and spending modalities varied were prominent in African countries. The establishment of such funds is meant to ensure an agile response to address existing delays and rigidities in public financial management systems (PFM) that would make it difficult to effectively respond to emergencies (especially where misalignments may impede the use of existing systems) [3]. Misalignments arise in systems that allocate budget by prescribed line items with minimal flexibility in the face of an emergency. In some countries, such flexibility is a legislative issue on parliamentary levels.

**Table 1:** Measures taken to finance COVID-19 response*

Measures taken	Countries
Established special funds for COVID-19 (28 countries)	Benin, Botswana, Burkina Faso, Cameroon, Chad, Comoros, Congo, Democratic Republic of Congo, Djibouti, Equatorial Guinea, Gabon, Ghana, Kenya, Lesotho, Mali, Mauritania, Mauritius, Nigeria, South Africa, Uganda, Zambia, Zimbabwe, Cote D'Ivoire, Liberia, Niger, Senegal, Togo, Tunisia
Re-allocation/supplementary budgets (14 countries)	Carbo Verde, Eswatini, Ethiopia, The Gambia, Ghana, Guinea Bissau, Madagascar, Mozambique, Namibia, Rwanda, Sao Tome Principe, Seychelles, United Republic of Tanzania, Uganda
Contingency/Reserve fund (4 countries)	Keya, Malawi, South Africa, Uganda
**Of the 26 special funds†**	
**Extra-budgetary (n = 22)**	**On budget (n = 4)**
Benin, Botswana, Cameroon, Democratic Republic of Congo, Cote D'Ivoire, Djibouti, Equatorial Guinea, Gabon, Ghana, Kenya, Lesotho, Liberia, Mali, Mauritania, Mauritius, Niger, Sierra Leone, South Africa, Togo, Tunisia, Uganda, Zimbabwe	Chad, Nigeria, Senegal, Zambia

*Source: The WHO COVID PFM database (The WHO COVID PFM database compiles information generated through a PFM web-survey designed and administered in April-May 2020 by WHO and other primary and secondary sources of information on: emergency spending measures, enactment of spending plans, formulation of spending plans, spending modalities and reporting mechanisms).

†Information on the nature of special funds for Mali and Tunisia was not available.

Additional reasons for creating such funds have been highlighted as an attempt to leverage high political control; in most cases, these funds were held at the presidential or governmental levels to enable the pooling of public and private resources to support response efforts, ensuring a whole of government approach in implementation and spending and separating COVID-19 spending with a distinct audit trail. Disbursed in lump sums, emergency funds are generally managed with limited, if any, transparency, accountability, or reviews, unlike standard programs funds [4]. Allen R cautions against turning these into “little empires” with limited transparency and accountability [5]. The International Monetary Fund (IMF) highlights potential risks, such as fragmenting policy making and clouding government’s fiscal discipline. Further, the IMF refers to “do what it takes but keep the receipts” as part of ensuring a prudent, transparent, accountable, and legitimate emergency response [6]. There is also a link between a legitimate/transparent response and public trust in responding to COVID-19.

While budgetary sources have mechanisms to ensure transparency and accountability through PFM, it is not clear if this applies to the extra-budgetary sources. Ideally, all raised funds should be part of a unified and comprehensive budget process, ensuring coherence and fiscal discipline in line with the country’s PFM practices (the lack of which is associated with many risks) [7]. Extra-budgetary funds carry large fiduciary risks if they are completely outside of the PFM system, are not subject to basic PFM rules, or have weak governance structures and institutions [8]. Zannath and Gurazada observe that, if COVID-19 emergency funds followed PFM rules, reporting on such expenditures would be more effective [9].

African countries have consistently scored lowest on the Corruption Perceptions Index (CPI). Hence, they require stronger systems that reduce vulnerabilities to corruption and misuse of funds to respond to the COVID-19 crisis. Worse still, emergency situations are fertile ground for vested interests to use public funds for private gain, making it important that vulnerability is not only recognized, but also mitigated [10].

**Figure:**
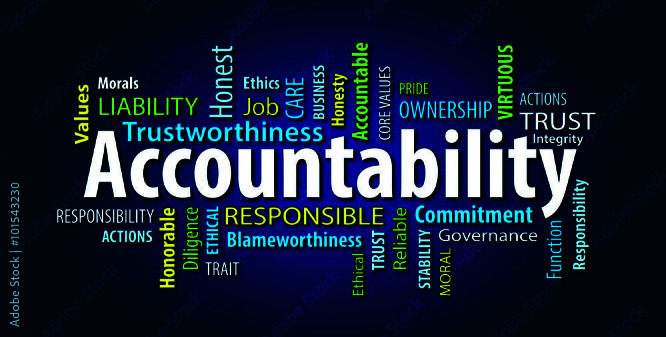
Photo: Source: Free Adobe Stock Image. Available at: https://stock.adobe.com/search?k=accountability&.

## COUNTRY EXPERIENCE ON ACCOUNTABILITY ARRANGEMENTS FOR EXTRA-BUDGETARY FUNDS

Building on the recommendations for the design/reform of COVID-19 extra-budgetary funds, we reviewed the mechanisms used to ensure their transparency and accountability in African countries [7]. We focused on three parameters, namely; the presence and nature of the management and oversight structures, and spending and accountability modalities.

The urgency of the COVID-19 pandemic required swift action and a quick allocation and spending of funds, which results in a relaxation of the standards procedures. Given the unprecedented scale of this health crisis as well as the financial resources required to respond to it, a rather more scrupulous and scrutinous oversight and accountability framework needs to be in place [11] because of the important incentive for corruption and theft of public money this crisis creates [12].

Fourteen out of the 22 African countries that allocated extra-budgetary COVID-19 funds did not provide any information on at least one of the three parameters analyzed. The management and oversight of COVID-19 extra-budgetary funds were held my ministries of health and the treasury in only three countries (Benin, Botswana, and Djibouti). Eleven countries employed ad hoc measures, including setting up independent committees and structures to manage COVID-19 funds.

The dramatic increase in the amounts and speed of spending created by the COVID-19 crisis, as well as distractions or standards relaxation hindering oversight mechanisms created incentive for corruption [1,2]. Among the 14 African countries, the lack of explicit transparency and weak oversight could exacerbate fraud and mismanagement of these public funds [13], deeply impacting public trust [14]. According to IMF, less than a year into the pandemic, dozens of corruption media reports and other criminal activities related to COVID-19 spending were unveiled [4].

The lack of information around the management and oversight of these public funds and accounting mechanisms poses a major threat to their effective use. Stakeholders such as civil society organizations and development partners (besides the entities assigned the management of these funds) have a limited role in monitoring the spending of these public funds, which negatively impacts their transparency and accountability [15,16].

A study conducted in Nigeria [16] showed that the lack of transparency and accountability around COVID-19 funds was found to be a factor for political distrust which, in turn, undermines the government response to the pandemic. Generally speaking, tolerating malfeasance and poor management of funds hurts long-term government legitimacy and helps increase the spread of virus among the most vulnerable [13]. Therefore, putting in places accountability mechanisms for public funds not only helps manage resources better, but also contributes substantially to increasing public compliance to the government response efforts and building public trust, especially in low- and middle-income countries [16,17], where corruption stands between an effective collaboration between the government and the public. The COVID-19 pandemic, with its unprecedent scale, should be an opportunity to re-establish public trust in governments that have long carried a legacy of mismanagement and corruption [17].

Turning to spending modalities, in ten (Benin, Cameroon, Mali, Mauritania, Niger, Sierra Leone, Togo, Tunisia, Uganda, and Zimbabwe) out of 22 countries, the areas/interventions to be supported by the COVID-19 special funds were not stated. In some countries (Kenya, Liberia, Botswana), the areas to be funded using these funds were broad and multiple. Several questions arise regarding the focus of these funds – should they complement available funding, ie, focus on funding gaps/unfunded areas? Should they seek to harness available opportunities that are not routinely incorporated in government funding? Should they do both, depending on the context? While the former seeks to minimize duplications in funding (requiring the supported areas to be explicit), the latter seeks to ensure that non-traditional sources (in the case of Africa, the private sector) are pooled into the overall response. Although we did not undertake an extensive review of the allocation patterns to analyse the areas and interventions funded by the different sources, duplications are visible in some of the countries (DRC, Djibouti, Kenya, Lesotho, Liberia, Mauritius) where funds were reportedly allocated to “routine response interventions” which should ideally be funded from government budget sources. The focused support in the utilization of these funds in Equatorial Guinea, Gabon and South Africa is especially noteworthy. Ghana presents an example of pooling private sources of funding into the overall response.

Zannath and Gurazada [9] elaborate key principle that warrants consideration in setting up emergency funds. These include the need to ensure complementarity between the different funding streams; enhancing and communicating protocols to minimize waste, fraud and corruption; establishing reporting arrangements that link outputs and outcomes to availed funds; enhancing oversight credibility through the involvement of supreme audit institutions or a credible external audit firm and; civil society playing a watchdog role. The World Bank [18] on the other hand, underscores simplicity in the systems; discretions to ensure agility whilst ensuring transparency and accountability; creating room for the public and civil society to monitor the use of resources; laying emphasis on areas with potential risk eg, economic support packages; communicating and enforcing anti-corruption measures and; implementing review mechanisms ‘keeping an eye on the rearview mirror’ alongside response efforts.

## CONSIDERATIONS IN BUILDING BACK BETTER

### Building strong institutions

The principles detailed by Zannath and Gurazada [9] and the World Bank Group [18] stress a need for strong institutions capable of developing comprehensive plans, ensuring coherence in implementation, undertaking review and accountability and establishing structures that engender participatory processes. Facing such a large crisis where the funds can come from multiple sources, governments and other stakeholders must collaborate to streamline and synergize all efforts for better effectiveness and efficiency [14]. The COVID-19 pandemic is a reminder that building institutional and systems capacity should be a continuous national and international effort to sustain the delivery of better health outcomes during and after the pandemic [19].

### Strengthening government legitimacy

The Nigeria example highlights how lack of transparency and accountability around COVID-19 funds contributed to political mistrust [16] which can negatively impact the uptake of interventions. A major consideration in addressing this challenge is enhancing transparency through timely sharing of information, protocols, and expenditure data. Governments need to exploit the opportunities offered by digital technologies to share information, mobilize communities, and ensure meaningful participation of stakeholders. Digital technologies such as business intelligence tools could also be used to monitor COVID-19 funds. Funding- and program-related information should be made public and accessible online as a sign of the governments’ continued commitment to good governance and transparency principles [4].

Risks such as collusion, overpricing, and hidden contracts need to be mitigated. Public contracts and all the information related to the companies awarded contracts (including payments) need to be publicized. Open measures allow for stronger oversight mechanisms through greater reliance on bottom-up social accountability, while public participation in monitoring and exercising accountability help expose and reduce corruption [4]. While encouraging citizens’ active participation in such accountability and transparency efforts, it is important to consider whistle blower protection to avoid fear of retaliation [4,14,19].

### Build resilient public financial management systems

We draw from the work of Gupta et al. [3] who advocate for inbuilt flexibility that allows for swift reallocation, response plans, and interventions. Equally important is the need to stipulate clear roles in budgeting (especially in decentralized settings) and modalities to ensure the accelerated release of public funds. The key consideration in these arguments hinges on building agile PFM systems that serve to manage financial processes of governments, including emergency response settings.

## POLICY RECOMMENDATIONS AND CONCLUSIONS

Despite the enormous burden on countries to plan, respond, and recover from the current pandemic, emphasizing efficient oversight mechanisms is paramount for a successful response. While the need to relax standard PFM measures is expected, this should not mean that basic accountability and transparency principles cannot be established. This is particularly important for African countries because of the history of fragility of institutional bureaucracy and the public distrust in governments. We underscore the need for strong institutions and PFM systems that are adaptable to shocks. Despite the great challenges it brings, this unprecedented crisis offers an opportunity towards re-establishing trust between governments and communities, creating a historic momentum and a space that fosters cross-sector and multi-stakeholder collaboration for a more streamlined and efficient use of public resources. This serves to empower citizens and enhance their participation in good governance to share the burden on public institutions.
